# How Do MinC-D Copolymers Act on Z-Ring Localization Regulation? A New Model of *Bacillus subtilis* Min System

**DOI:** 10.3389/fmicb.2022.841171

**Published:** 2022-04-15

**Authors:** Na Wang, Tingting Zhang, Shuheng Du, Yao Zhou, Yaodong Chen

**Affiliations:** ^1^Key Laboratory of Resources Biology and Biotechnology in Western China, Ministry of Education, College of Life Sciences, Northwest University, Xi’an, China; ^2^Provincial Key Laboratory of Biotechnology of Shaanxi Province, Northwest University, Xi’an, China

**Keywords:** bacterial cell division, MinC, MinD, MinC-D copolymers, Z-Ring, FtsZ

## Abstract

Division site selection in rod-shaped bacteria is strictly regulated spatially by the Min system. Although many sophisticated studies, including *in vitro* recombination, have tried to explain these regulations, the precise mechanisms are still unclear. A previous model suggested that the concentration gradient of MinC, an FtsZ inhibitor, regulates the position of the Z-ring in the cell. In *Escherichia coli*, the oscillation of MinCDE proteins leads to a gradient of Min proteins with the average concentration being lowest in the middle and highest near the poles. In contrast to the Min system of *E. coli*, the Min system of *Bacillus subtilis* lacks MinE and exhibits a stable concentration distribution, which is regulated by the binding of DivIVA to the negative curvature membrane. The Min proteins first accumulate at the poles of the cell and relocalize near the division site when the membrane invagination begins. It is inconsistent with the previous model of high concentrations of MinC inhibiting Z-ring formation. Our preliminary data here using electron microscopy and light scattering technology reported that *B. subtilis* MinC (BsMinC) and MinD (BsMinD) also assembled into large straight copolymers in the presence of ATP, similar to the Min proteins of *E. coli*. Their assembly is fast and dominated by MinD concentration. When BsMinD is 5 μM, a clear light scattering signal can be observed even at 0.3 μM BsMinC. Here, we propose a new model based on the MinC-D copolymers. In our hypothesis, it is not the concentration gradient of MinC, but the MinC-D copolymer assembled in the region of high concentration MinD that plays a key role in the regulation of Z-ring positioning. In *B. subtilis*, the regions with high MinD concentration are initially at both ends of the cell and then appear at midcell when cell division began. MinC-D copolymer will polymerize and form a complex with MinJ and DivIVA. These complexes capture FtsZ protofilaments to prevent their diffusion away from the midcell and narrow the Z-ring in the middle of the cell.

## Introduction

Bacterial cell division is initiated by a dynamic Z-ring, which consists of the self-assembled FtsZ protofilaments and dozens of associate proteins (Haeusser and Margolin, 2016; Erickson and Osawa, 2017; McQuillen and Xiao, 2020). FtsZ, a bacterial tubulin homolog, is the key protein for bacterial division. In *Escherichia coli*, FtsZ and its membrane-tethering proteins ZipA and FtsA first form a proto-ring and then act as a scaffold to recruit dozens of downstream proteins to form a mature ring. FtsZ protofilament, as the most basic component of the Z-ring, exhibits very dynamic characteristics *in vitro* and *in vivo* (Stricker et al., 2002; Chen and Erickson, 2005, 2009). Recent studies revealed that FtsZ filaments or bundles exhibited treadmilling dynamics *in vivo*, traveling along the path of the Z-ring, selectively adding FtsZ subunits to one end, and releasing from another end (Bisson-Filho et al., 2017; Yang et al., 2017; Ramirez-Diaz et al., 2018; McCausland et al., 2021).

The bacterial division is strictly regulated spatially and temporally by a variety of regulatory proteins. The dynamic Z-ring in rod-shaped bacteria locates precisely in the center of the cell and is regulated by at least two negative regulation systems: the Min system and nucleoid occlusion (NO) system (Lutkenhaus and Du, 2017; Schumacher, 2017; Szwedziak and Ghosal, 2017). NO system, mediated by Noc in *Bacillus subtilis* or SlmA in *E. coli*, prevents bacteria from dividing over the chromosome. Meanwhile, the Min system prevents Z-ring assembly near the poles.

### Current Model Suggests a Concentration Gradient of MinC in the Cell Regulates the Z-Ring Position. High Concentration of MinC Inhibits FtsZ Assembly to Prevent the Assembly of the Z Ring Near the Pole

**FIGURE 1:**
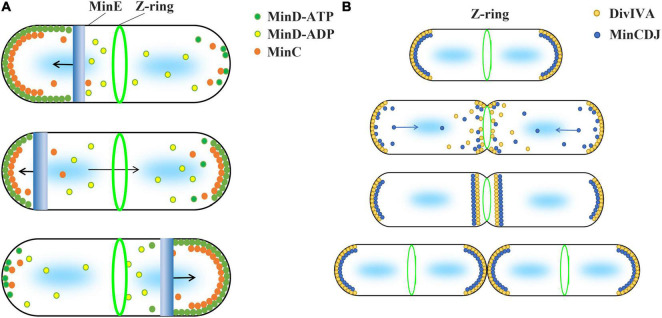
Current model suggests that the concentration gradient of MinC in cell regulates the Z-ring position. The two types of Min system use different strategies to form protein concentration gradients. **(A)** The Min system of Gram-negative bacteria, such as *E. coli* and *P. aeruginosa*, consists of MinC, MinD, and MinE proteins and is known as the MinCDE system. The MinD-ATP dimer (green) preferentially binds to the membrane, while the MinD-ADP subunits (yellow) detach from the membrane. MinE (blue) can stimulate the ATPase activity of MinD and release MinD and MinE from the membrane. This makes MinE drive the oscillation of MinD from one end of the cell to the other, which leads to an apparent concentration gradient of MinD in the cell, with the average concentration being lowest in the middle of the cell and highest near the poles. MinC (orange), as an FtsZ inhibitor, binds to MinD and moves with the movement of MinD. High concentration of MinC inhibits FtsZ polymerization, which is used by current models to explain how the Min system regulates the positioning of Z-ring. **(B)** Gram-positive bacteria, such as *B. subtilis*, contain MinC, MinD, MinJ, and DivIVA proteins known as the MinCDJ system. Min proteins (blue) concentration gradient is regulated by DivIVA (orange), which favors attaching to the negative curvature membrane. When DivIVA is mainly bound at both ends of the cell, MinD exhibits a stationary bipolar gradient pattern. This is easily explained by current models. However, DivIVA proteins will relocalize at midcell as soon as the membrane invagination begins, and MinCDJ proteins localize to division sites and poles at the same time in dividing cells. It cannot be explained by the existing model that high concentration MinC inhibits the assembly of Z-ring. A revised model suggested that MinCDJ moved to the middle of the cell to prevent a new round of division and/or might act on some downstream proteins of Z-ring assembly.

Min systems are mainly summarized into two different types, MinCDE and MinCDJ, which have been well studied in Gram-negative bacteria *E. coli* and Gram-positive bacteria *B. subtilis*, respectively (Lutkenhaus and Du, 2017; Szwedziak and Ghosal, 2017). In general, the current inhibition model uses the uneven distribution of Min proteins and the inhibitory effect of MinC on FtsZ polymerization to explain why Z-ring is accurately located in the middle of the cell (Figure 1). This model is based on two important facts. First, MinD protein is unevenly distributed in cells. Second, MinC directly interacts with FtsZ and inhibits FtsZ polymerization.

Both MinC and MinD are the key proteins and exist in both systems. MinD is an ATPase with a deviant walker A motif (Lutkenhaus and Sundaramoorthy, 2003). ATP-bound MinD tends to form a dimer and prefers to bind to the membrane by its C-terminal amphipathic helices. MinC, as an FtsZ inhibitor, is the main component in the Min system to interact with FtsZ directly. The previous biochemical results showed that MinC inhibited the polymerization of FtsZ, but did not change the GTP hydrolysis activity of FtsZ (Hu et al., 1999). Subsequent results suggest that MinC may only shorten the length of FtsZ protofilaments, but does not affect the amount of FtsZ subunits in the filaments (Dajkovic et al., 2008; Shen and Lutkenhaus, 2010; Hernandez-Rocamora et al., 2013; Huang et al., 2018).

MinC has two distinct domains of similar size. Both domains are necessary for their physiological function to regulate the Z-ring position (Hu and Lutkenhaus, 2000). MinC C-terminal domain (MinCC) leads MinC to form a dimer and binds both MinD and C-terminal of FtsZ. However, MinC N-terminal domain (MinCN) binds to FtsZ and inhibits FtsZ assembly (Hu and Lutkenhaus, 2000; Cordell et al., 2001; Shiomi and Margolin, 2007; Shen and Lutkenhaus, 2009, 2010). It is proposed that MinCC is first connected to the C-tail of FtsZ, and then, MinCN attacks the H10 helix of FtsZ to break the FtsZ protofilament without affecting the GTPase significantly (Shen and Lutkenhaus, 2010).

MinCDE system mainly contains MinC, MinD, and MinE proteins. The previous studies showed that the MinCDE system in *E. coli* displayed an oscillating pattern; the proteins moved quickly from one end of the cell to the other, completing a cycle in ∼50 s (Hu and Lutkenhaus, 1999; Raskin and De Boer, 1999). The oscillation is mainly caused by a feedback loop of the ATPase MinD and its activator MinE (Loose et al., 2008; Park et al., 2011, 2017; Arumugam et al., 2014; Denk et al., 2018). The accumulation of MinE activates the ATPase activity of the MinD bound to the membrane, converting MinD from dimers to monomers and releasing them from the membrane. The oscillation of MinDE proteins leads to a gradient of MinD in the cell, with the average concentration being lowest in the middle of the cell and highest near the poles.

The previous results considered that only MinC of Min system directly interacted with FtsZ and inhibited the Z-ring formation (Hu et al., 1999). As a passenger, MinC combines with MinD and moves with MinD to form a concentration gradient. In the high concentration MinC region, FtsZ polymerization is inhibited, thereby preventing the assembly of Z-ring.

However, MinC is only a weak inhibitor of FtsZ. The previous biochemical results suggested that only a high concentration of MinC could shorten the length of FtsZ protofilaments (Scheffers, 2008; Huang et al., 2018) or reduce the FtsZ bundles (Dajkovic et al., 2008). Moreover, MinC concentration *in vivo* is pretty low. In *E. coli*, the previous reports revealed that the average MinC concentration was around 0.7 μM, which was 6–8 times less than that of FtsZ and MinD (Li et al., 2014; Erickson and Osawa, 2017). So, how does the small amount of MinC *in vivo* effectively regulate the Z-ring position? Although the existing inhibition models emphasize that MinC concentration *in vivo* varies with MinD concentration, it is still difficult to explain why MinC concentration is so low.

MinCDJ system includes at least four proteins, that is, MinC, MinD, MinJ, and DivIVA. In contrast to the oscillating MinCDE system, MinCDJ proteins are considered to exhibit a stationary bipolar gradient pattern, which is regulated by DivIVA (Cha and Stewart, 1997; Edwards and Errington, 1997). DivIVA favors attaching to the negative curvature membrane (Lenarcic et al., 2009) and accumulating at the pole region of the cell at the early stage. After recruiting the transmembrane protein MinJ as a bridge, the DivIVA/MinJ complex binds to MinD to regulate the position of MinD (Marston et al., 1998; Patrick and Kearns, 2008), which forms a rather stable concentration gradient with the highest at the poles and lowest in the middle to allow the Z-ring assembly at midcell. However, further studies discovered that part of the DivIVA protein would relocalize later near the division site as soon as the membrane invagination begins, and MinCDJ proteins preferentially localized to division sites and poles at the same time in dividing cells (Bramkamp et al., 2008; Gregory et al., 2008; van Baarle and Bramkamp, 2010; Eswaramoorthy et al., 2011; Bach et al., 2014). The accumulation of high concentrations of MinC at midcell in *B. subtilis* is still elusive, because it is inconsistent with the current model of high concentration of MinC inhibiting FtsZ assembly. The alternative model suggested that MinCDJ moved to the middle of the cell to prevent a new round of division and/or might act on downstream of Z-ring assembly (Bramkamp and van Baarle, 2009).

In summary, several experimental results contradict the model that high concentration of MinC near the poles inhibits FtsZ polymerization, so that Z-ring cannot assemble outside the middle of the cell. Specifically, in *B. subtilis*, MinC proteins gather not only at both ends of the cell, but also in the middle as soon as the beginning of cell division. The convergence of MinC protein in the middle of the cell is contradictory to the inhibition hypothesis. An alternative model attempts to explain this phenomenon, speculating that MinC might act on other unknown downstream proteins. But this explanation is not satisfactory. Also, the concentration of MinC in *E. coli*, as well as possibly in other bacteria, is much lower than that of FtsZ. It is difficult to explain with the existing assumptions about MinC concentration gradients.

### MinC-D Copolymers in the MinCDE Systems Greatly Enhance the Binding Coefficient Between MinC and FtsZ

The formation of MinC-D copolymers can explain directly why MinC concentration is very low. The previous reports revealed that MinC and MinD from *E. coli* and *Pseudomonas aeruginosa* coassembled into long filaments with 1:1 stoichiometry and a MinC_2_-MinD_2_-MinC_2_-MinD_2_ pattern in the presence of ATP (Ghosal et al., 2014; Conti et al., 2015; Huang et al., 2018). These copolymers’ assembly was mainly dominated by the concentration of MinD protein, and if MinD concentration was high enough (>4 μM for *P. aeruginosa*), a very low concentration of MinC could coassemble with MinD to form copolymers (Huang et al., 2018). It is consistent with the protein concentration *in vivo*, in which the MinC concentration is around 0.7 μM, only 1/6 of MinD (Erickson and Osawa, 2017). Due to the multi-sites binding between MinC-D copolymers and FtsZ protofilaments, this greatly enhances the binding coefficient between MinC and FtsZ, which can explain why the low concentration of MinC *in vivo* can also function on FtsZ (Ghosal et al., 2014; Huang et al., 2018).

MinE not only removed MinD from the membrane but also disassembled MinC-D copolymers (Ghosal et al., 2014; Huang et al., 2018). Due to the rapid MinE oscillation in the cell, MinC and MinD in the MinCDE system may only polymerize into short copolymers *in vivo*. The assembly–disassembly cycle and the movement of the short MinC-D polymers are regulated by MinE and MinD oscillation. Since MinD concentration is 6–8 times higher than that of MinC and MinE disassemble the MinC-D polymer, the copolymerization of MinC-D is likely not to affect the oscillation of MinD. MinC *in vivo* will copolymerize with MinD in the region of high concentration of MinD, which is regulated by MinDE oscillation. MinC-D copolymers capture FtsZ filaments and prevent them from diffusing to the poles of the cell. After that, MinC promotes the depolymerization of FtsZ protofilaments and FtsZ monomers are released for the next round.

### Our New Preliminary Results Show That MinC and MinD of *Bacillus subtilis* Can Also Coassemble to Form Similar Copolymers

There is no MinE in the MinCDJ system. MinD sequences among different bacterial species are well-conserved (Supplementary Figure 1), but MinC sequences are quite different (Supplementary Figure 2). The MinD sequence of *B. subtilis* and *P. aeruginosa* is 44% identical, whereas the MinC sequence is only about 20% identical. It is interesting to study whether or not MinC and MinD of the MinCDJ system can be copolymerized. If MinC-D of *B. subtilis* forms copolymer, they are likely to form large stable structures if BsMinC-D is difficult to depolymerize. During the division of *B. subtilis*, these structures will exist both at the poles and in the middle. To this end, we have purified *B. subtilis* MinC (BsMinC) and MinD (BsMinD) proteins and determined their assembly (the detailed methods are shown in Supplementary Document).

**FIGURE 2:**
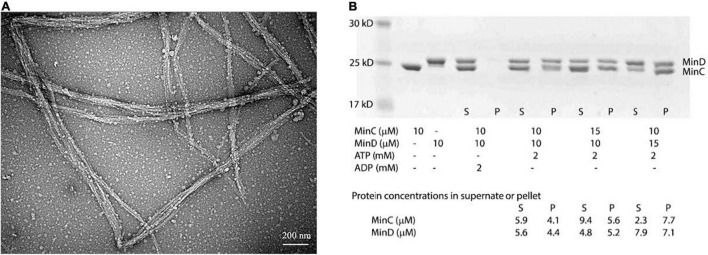
BsMinC and BsMinD coassembled into a long and large bundle in the ratio of 1:1. **(A)** Negative stain EM showed that 8 μM BsMinD and 4 μM BsMinC coassembled into large bundles in the presence of 2 mM ATP. Bar represents 200 nm. **(B)** SDS-PAGE analysis of cosedimentation in different ratios of BsMinC and BsMinD showed that the ratio of BsMinC and BsMinD in the pellet is always approximately 1:1. The initial concentration of the mixture, the estimated MinC and MinD concentrations in the supernatant and pellet are presented at the bottom of the panel. S, supernatant; P, pellet.

We first observed their assembly using the electron microscopy (EM)-negative stain technique. Incubating 8 μM BsMinD and 4 μM BsMinC with 2 mM ATP for 5 min, we observed long and large straight bundles composed of multiple filaments with a width of about 30–100 nm (Figure 2A), which were 5–8 times larger than the single- or double-strand MinC-D polymers of *P. aeruginosa* and *E. coli*. Sedimentation and SDS-PAGE analysis showed that the copolymer was composed of MinC and MinD in a ratio of 1:1 (Figure 2B). After centrifugation at 50,000 rpm, most copolymers were collected in the pellet. Although there are different initial concentrations of MinC and MinD, the ratio of MinC and MinD in the pellet is always approximately 1:1. As a control, only MinC or MinD alone (Supplementary Figure 3) and a mixture of MinC and MinD with ADP (Figure 2B) cannot form copolymers.

**FIGURE 3:**
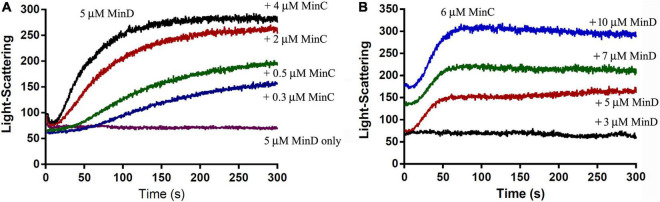
The comparison of the assembly kinetics of BsMinC and BsMinD at different concentrations suggested that BsMinD concentration dominated the coassembly of BsMinC-D. **(A)** Assembly kinetics of 5 μM BsMinD and different concentrations of BsMinC from 0.3 to 4 μM. **(B)** Assembly kinetics of 6 μM BsMinC and different concentrations of BsMinD from 3 to 10 μM. ATP concentration is 2 mM.

The electron microscope observation could not be well quantified, we next investigated the assembly properties of BsMinC-D copolymer with different protein concentrations and protein ratios using a light-scattering assay. An important conclusion of our previous studies on *P. aeruginosa* MinC-D assembly is that MinD protein dominated their polymerization, which is consistent with the concentration of these two proteins *in vivo* (Huang et al., 2018). We found similar results of BsMinC-D assembly. Figure 3 shows the assembly dynamics at different concentrations of BsMinC and BsMinD in the presence of 2 mM ATP. We observed that 5 μM BsMinD gave a detectable light scattering signal even at 0.3 μM BsMinC. Also, the polymerization accelerated when BsMinC concentration increased (Figure 3A). Meanwhile, when there was 6 μM BsMinC in the solution, only a slight polymerization would be observed when BsMinD concentration was at 3 μM (Figure 3B). It is suggested that BsMinC-D assembly requires a high concentration of BsMinD, and the critical concentration of BsMinD is around 3 μM. If MinD concentration is high enough, a very low concentration of MinC would join in the copolymers. It implies that MinC-D copolymers may be assembled only in the high concentration region of MinD *in vivo.*

### The Hypothesis That MinC-D Copolymer Is Involved in Z-Ring Position Regulation

Here, we reported that MinC and MinD of *B. subtilis* could also be copolymerized to form a large straight bundle. The previous reports of the copolymer formation of MinC-D from Gram-negative bacteria *E. coli*, *P. aeruginosa*, and *Aquifexaeolicus* (Ghosal et al., 2014; Huang et al., 2018), together with our results here, suggest that the polymerization of MinC-D copolymers may be widespread in both two types of bacterial Min systems. They have similar properties, and the MinD concentration dominates their polymerization. It suggests that MinC-D copolymers may be assembled only in the high concentration region of MinD *in vivo*.

We summarize two functions of MinC-D copolymers. The first is to increase the binding efficiency of MinC and FtsZ, so it greatly enhances the effect of MinC on FtsZ polymerization. Second, it can capture the diffused FtsZ protofilaments and prevent them from moving to both ends of the cell.

Based on the fact that in *B. subtilis*, MinC is also concentrated near the Z-ring in the middle of the cell, which is inconsistent with the model of MinC inhibiting the polymerization of FtsZ. Therefore, we propose a new model, which emphasizes that the capture of FtsZ protofilaments by MinC-D copolymer is the main regulation mode.

### A New Model for Bacterial Division Site Selection by Min System of *Bacillus subtilis*

Here, we purpose a new model to explain the regulation of Z-ring by Min proteins. Compared with the previous model, we believe that MinC forms copolymers with MinD in the area of high concentration of MinD, which prevents the diffusion of FtsZ filaments and accelerates the depolymerization of FtsZ, which is the key to regulation.

Our model also believes that the uneven distribution of MinD is the first step in regulation. MinD gradient is regulated by MinE in the MinCDE system and by DivIVA/MinJ in the MinCDJ system. From our results, both the coassemblies of MinC-D of *P. aeruginosa* and *B. subtilis* are dominated by the concentration of MinD protein. It is consistent with the concentration of Min proteins *in vivo* and it is suggested that MinC *in vivo* will coassemble with MinD in the area of high concentration of MinD.

**FIGURE 4:**
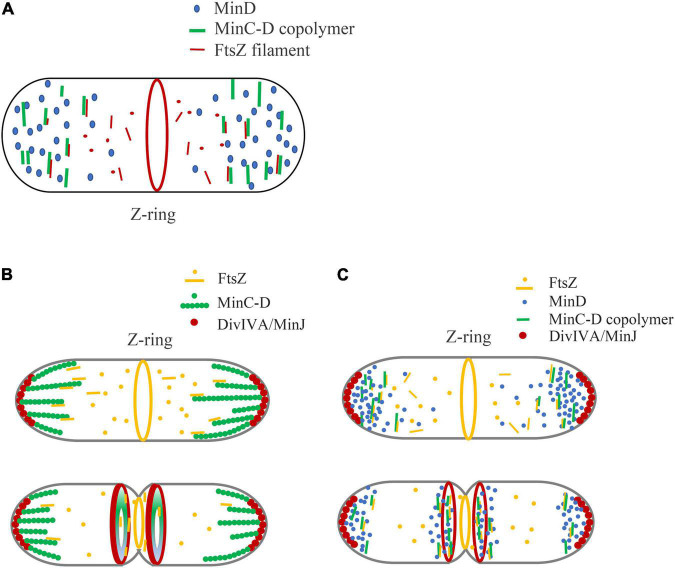
A new model is proposed to explain the regulation of Min system on Z-ring assembly positioning, emphasizing the role of MinC-D copolymer. **(A)** In general, we think that the binding of MinC-D copolymer to FtsZ protofilament is the key to Z-ring localization regulation. MinC will coassemble with MinD in the area of high concentration of MinD. The tight binding between MinC-D copolymers and FtsZ filaments not only greatly enhances the binding coefficient between MinC and FtsZ subunits, but also can capture FtsZ protofilaments, and prevent their diffusion to the ends of the cell. This may occur in both MinCDE and MinCDJ systems. **(B,C)** Show our new model of how Min proteins regulate Z-ring position in *B. subtilis*, if Min proteins form stable structures **(B)** or dynamic structures **(C)**. Whether MinC/MinD/DivIVA/MinJ complex is stable or dynamic *in vivo* is controversial. The location of DivIVA determines the uneven distribution of MinD proteins: at the early stage of division, MinD is only located at both ends of the cell, and when membrane invagination begins, MinD accumulates both at the cell poles and midcell. MinC-D copolymers are formed in the area of high concentration of MinD, and form a relatively stable complex **(B)** or dynamic complex **(C)** with DivIVA and MinJ. If the complex is dynamic, MinC-D copolymers can be released from DivIVA and accumulated near DivIVA/MinJ complex. This protein complex, when located at the ends of the cell, prevents FtsZ from diffusing to the ends of the cell. And when it is in the middle, this complex can further restrict the Z-ring to a narrow area in the middle. After the MinC-D copolymers and FtsZ protofilaments are tightly combined, MinC accelerates the depolymerization of FtsZ protofilaments.

Figure 4 shows our model of the inhibitory mechanism of Min system in *B. subtilis*, based on the role of MinC-D copolymers. Due to the multi-site binding between MinCD copolymer and FtsZ profilament, the binding efficiency of MinC and FtsZ is greatly enhanced, which can capture the diffused FtsZ profilament and strengthen the influence of MinC on FtsZ polymerization. This works on both MinCDE and MinCDJ systems (Figure 4A).

Figures 4B,C show the regulation of Z-Ring by *B. subtilis* Min protein. In the early stages of cell division of *B. subtilis*, DivIVA mainly binds to the curved membrane areas at both ends of the cell, and this makes MinD accumulate at the cell poles to form a bipolar gradient. MinC will coassemble with MinD at both ends of the cell, preventing the diffusion of FtsZ from leaving the middle of the cell. When membrane invagination begins, part of DivIVA will relocate to the curved membrane area at midcell next to the Z-ring and cause the DivIVA/MinJ/MinC-D complex to form on both sides of the Z-ring. MinC-D complex prevents FtsZ from diffusing and restricts the Z-ring to a narrow area in the middle. After the MinC-D copolymers and FtsZ protofilaments are tightly bound, MinC accelerates the depolymerization of FtsZ protofilaments. Then, the MinC-D copolymers will release FtsZ monomers and recapture the new diffusing FtsZ protofilaments.

The difference between Figures 4B,C is mainly to consider whether MinC/MinD/DivIVA/MinJ will form a stable large complex. Without MinE in the MinCDJ system, it seems that MinC and MinD will assemble into a stable large bundle and form a stable complex with MinJ and DivIVA *in vivo*. Eswaramoorthy et al. (2011) reported that two stable adjacent rings of *B. subtilis* Min proteins were assembled on both sides of the Z-ring during cytokinesis, which is an evidence that they form a stable structure. However, Feddersen et al. (2021) had recently observed using the FRAP technique that Min proteins in *B. subtilis* were also dynamic. It is reported that MinD, located in the middle of the cell, as well as MinJ and DivIVA, also showed high dynamic characteristics. How is the dynamics of Min protein regulated? Is MinC-D complex also dynamic *in vivo*? Does this dynamic help the rapid depolymerization of MinC-D-FtsZ complex to quickly release the bound FtsZ subunits? Is their regulation related to DivIVA and MinJ, or to other unknown proteins? These are problems that need to be addressed.

## Summary

In conclusion, we propose a new model of Min protein regulation that emphasizes the role of MinC-D copolymers in this article. In our model, the key point is that we think that it is not the concentration gradient of MinC, but the MinC-D copolymer formed in the region of high concentration MinD that plays a key role in the regulation of Z-ring positioning. In general, the binding of MinC-D copolymer with FtsZ protofilament greatly enhances the binding efficiency of MinC and FtsZ, which not only accelerated the depolymerization of FtsZ but also prevented the diffusion of FtsZ filaments to the cell ends.

In the MinCDE system, our model is not significantly different from the existing models. Since MinC-D copolymer can be depolymerized by MinE protein, and the number of MinD *in vivo* is more than 6 times that of MinC, MinE-driven MinD oscillation may not be affected by the copolymerization of MinC-D. The role of MinC-D copolymer is to greatly increase the binding coefficient of MinC and FtsZ. The regulation of Z-Ring position can be interpreted as MinC inhibits the polymerization of FtsZ, or MinC-D copolymer prevents the diffusion of FtsZ filaments.

However, in the MinCDJ system, the existing inhibition model is inconsistent with the experimental results. MinC and MinD proteins in B. subtilis accumulate at both ends of cells, but also in the middle of cells when cell division begins. Thus, our new model can more directly explain its regulation. The MinC/MinD/DivIVA/MinJ complexes form near the Z-ring, preventing FtsZ from spreading out of the middle of the cell and narrowing the contraction ring.

## Data Availability Statement

The original contributions presented in the study are included in the article/Supplementary Material, further inquiries can be directed to the corresponding author/s.

## Author Contributions

NW, TZ, and YC did most of the experimental work and interpretation. SD and YZ contributed experimental work and interpretation. YC conceived the project and wrote the manuscript with contributions from all authors. All authors reviewed the results and approved the final version of the manuscript.

## Conflict of Interest

The authors declare that the research was conducted in the absence of any commercial or financial relationships that could be construed as a potential conflict of interest.

## Publisher’s Note

All claims expressed in this article are solely those of the authors and do not necessarily represent those of their affiliated organizations, or those of the publisher, the editors and the reviewers. Any product that may be evaluated in this article, or claim that may be made by its manufacturer, is not guaranteed or endorsed by the publisher.

## Abbreviations

EM: electron microscopy
BsMinC: MinC of *Bacillus subtilis*
BsMinD: MinD of *Bacillus subtilis*
PaMinC: MinC of *Pseudomonas aeruginosa*
PaMinD: MinD of *Pseudomonas aeruginosa.*
