# Supplementary material for: How Do MinC-D Copolymers Act on Z-Ring Localization Regulation? A New Model of Bacillus subtilis Min System
Source: Front Microbiol. 2022 Apr 15;13:841171. doi: 10.3389/fmicb.2022.841171 (PMC9051478; doi:10.3389/fmicb.2022.841171)
Supplement: Supplementary file 1 [file Data_Sheet_1.PDF]

Supplementary Documents

How do MinC-D copolymers act on Z-ring localization regulation? A new model of *Bacillus subtilis* Min system

EXPERIMENTAL PROCEDURES

Plasmid construction and protein purification

Expression vectors for *B. subtilis* MinC, MinD, and C-terminus truncated MinD mutant 1-245 were constructed in the plasmid pET15b at the NdeI/BamHI sites. Since the full length of MinD Protein has expression and solubility problems, we used C-terminus truncated MinD protein in most experiments. His6-tag proteins were expressed in *E. coli* BL21 at 16°C overnight by the addition of 0.5 mM isopropyl β-D-1-thiogalactopyranoside (IPTG). Following sonication and centrifugation, the soluble His6-tag proteins were purified by affinity chromatography on a Talon column (Clontech Lab, Inc.). Proteins were firstly washed with 0-30 mM imidazole and then eluted with the elution buffer containing 50 mM Tris pH 7.7, 300 mM KCl, 300 mM imidazole. After dialysis with HMK72 buffer (50 mM HEPES, pH 7.2, 5 mM MgAc, 100 mM KAc), proteins with His6 tag were stored at −80 °C.

P. aeruginosa MinC and MinD proteins were purified as described previously (Huang et al., 2018) and proteins were stored at −80 °C.

Light-scattering measurement

The kinetics of MinC-MinD assembly was measured using a light scattering assay as described previously(Huang et al., 2018). After the appropriate concentration MinC-MinD mixture was quickly mixed with ATP, the light-scattering signal began to be measured. The Light scattering was measured using a Shimadzu RF-5301 PC spectrofluorometer, with both excitation and emission at 350 nm. Each measurement was repeated two or three times.

Electron Microscopy (EM)

The copolymer of MinC-D was visualized by negative stain EM. About 10 µL of the appropriate concentration protein mixture and 2 mM ATP were incubated at room temperature for several minutes and then applied to a carbon-coated 400 mesh copper grid. After 15 s, samples were

dried with filter paper and stained with 2% uranyl acetate for 10 s. Then, the excess droplets were absorbed with filter paper and air dried. The grids were saved and used immediately for imaging. Images were performed on a Hitachi H-7650 electron microscope.

Sedimentation assay

Sedimentation assay was used to determine the quantitative ratio of MinC and MinD in the copolymer. MinC and MinD at different concentrations were assembled with 2 mM ATP or ADP at room temperature for 10 min. After centrifuged at 50,000 rpm for 25 min at 20 °C in a Beckman TLA100 rotor, the supernatant was carefully removed with a pipette, and the pellet was resuspended in the same volume of the solution. By analyzing the protein in the pellet and supernatant by SDS-polyacrylamide gel electrophoresis (SDS-PAGE), the ratio of supernatant and pellet was quantified using ImageJ software, and the protein concentrations were calculated from the percentage of total protein concentration.

REFERENCE

Huang, H., Wang, P., Bian, L., Osawa, M., Erickson, H.P., and Chen, Y. (2018). The cell division protein MinD from Pseudomonas aeruginosa dominates the assembly of the MinC-MinD copolymers. *J Biol Chem* 293(20), 7786-7795. doi: 10.1074/jbc.RA117.001513.
