# Supplementary material for: How Do MinC-D Copolymers Act on Z-Ring Localization Regulation? A New Model of Bacillus subtilis Min System
Source: Front Microbiol. 2022 Apr 15;13:841171. doi: 10.3389/fmicb.2022.841171 (PMC9051478; doi:10.3389/fmicb.2022.841171)
Supplement: Supplementary file 2 [file Data_Sheet_2.pdf]

A

```
E. coli          mariivvtsgkggvgkttssaaiatglaqkgkktvvidfdiglrnldlimgcerrvvydf 60
K. pneumoniae    mariivvtsgkggvgkttssaaiatglaqkgkktvvidfdiglrnldlimgcerrvvydf 60
P. aeruginosa    makilvvtsgkggvgktttsaaigtglalrgfktvivdfdvglrnldlimgcerrvvydf 60
B. subtilis      mgeaivitsgkggvgktttsanlgtalailgkrvclvdtdiglrnldvvmglenriiydl 60
Listeria         mgeaivitsgkggvgkttstanlgtalalqgkkvclidmdiglrnldvvlglenriiydl 60
M. tuberculosis  maeiivvtsgkggvgkttssaslacglarrgkkvavidfdvglrnldlimgcerrvvydf 60
H. pylori        maivvtitsgkggvgkstttanlaiglaesgkkvvavdfdiglrnldmilglenrivfdv 60
                 *.  :.:*********:*::* :. .**  * :.  :* *:******:::* *.*:::*.

E. coli          vnviqgdatlnqalikdkrtenl-yilpasqtrdkdaltregvakvlddlkamdfefivc 119
K. pneumoniae    vnviqgdatlnqalikdkrtenl-yilpasqtrdkdaltregvdkvleelkkmefdfivc 119
P. aeruginosa    vnvvngeatltqalikdkrlenl-hvlaasqtrdkdaltkegvekvmaelr-kdfeyiic 118
B. subtilis      vdvvegrckmhqalvkdkrfddllylmpaaqtsdktavapeqiknmvqelk-qefdyvii 119
Listeria         vdvvegrckihqamikdkrfddllfllpaaqttdknavsgeqmielinqlr-pdydyili 119
M. tuberculosis  vnvvhgeatlkqalikdkrfdnl-yvlaasqtrdkdaltqegvrkvlddliadgfeyvic 119
H. pylori        vdvmekncnlsqalitdkktknl-sflaasqskdknildkekvailinalr-vdfdyili 118
                 *:*:.  ..: **::.**: .:*  .: *:*: **  :  * :  ::  *    :::::

E. coli          dspagietgalmalyfadeaiittnpevssvrdsdrilgilasksrraengeepikehll 179
K. pneumoniae    dspagietgalmalyfadeaiittnpevssvrdsdrilgilasksrraengeepikehll 179
P. aeruginosa    dspagiekgahlamyfadeaivvtnpevssvrdsdrmlgllasksqraekgeepikehll 178
B. subtilis      dcpagieqgyknavsgadkaivvttpeisavrdadriiglleqe-------enieepprlv 172
Listeria         dcpagietgyknavagadkaivvttpeisavrdadriigllek--------edieppkli 171
M. tuberculosis  dspagiekgaflamyyadravvvvnpevssvrdsdriiglldskthkaesgkn-vpafll 178
H. pylori        dspagiesgfehailhadmalvvvtpevsslrdsdrvigiidaksnrakrgee-vhkhli 177
                 *.***** *   *:  ** *::...**:*::**:**::*::          ::     *:

E. coli          ltrynpgrvsrgdmlsmedvleilriklvgvipedqsvlrasnqgepvildinadagkay 239
K. pneumoniae    ltrynpgrvnkgdmlsmedvleilrinlvgvipedqsvlrasnqgepvildaasdagkay 239
P. aeruginosa    ltrynpervtkgemlgvddveeilairllgvipesqavlkasnqgvpvildeqsdagqay 238
B. subtilis      vnrirnhlmkngdtmdideivqhlsidllgivadddevikasnhgepiamdpknrasiay 232
Listeria         inrirtqmmvngdvmdideitthlsiellgiiidddevirssnsgdpvamlpnnrasqgy 231
M. tuberculosis  ltryspvrvesgemlsitdveevlglkaigvvpesgdvlnasnkgepvildpesaagqay 238
H. pylori        inrlkpelvangemisieevlkilclpligiipedshiisatnkgepvirt-dcesakay 236
                 :.*     :  *: :.: ::   * :  :*:: :.  :: ::* * *:       :. .*

E. coli          adtverllgeerpfrfieeekkgflkrlfgg----- 270
K. pneumoniae    adtverllgeerpfrfieeekkgflkrlfgg----- 270
P. aeruginosa    sdavdrllgkeiphrfldvqkkgflqrlfggre--- 271
B. subtilis      rniarrilgesvplqvleeqnkgmmakiksffgvrs 268
Listeria         rniarrilgesiplmsieakkpgfftrlkqlfggk- 266
M. tuberculosis  edavgrilgeerpmrfttvekkgffsklfgg----- 269
H. pylori        qritrrilgeeveyvefk-akrgffgalkgifs--- 268
                    . *:**:.           : *::  :
```

B

E. coli	100. 00	95. 56	75. 09	43. 13	45. 21	69. 14	50. 00
K. pneumoniae	95. 56	100. 00	75. 46	44. 27	44. 83	67. 66	49. 62
P. aeruginosa	75. 09	75. 46	100. 00	43. 56	43. 73	70. 52	48. 51
B. subtilis	43. 13	44. 27	43. 56	100. 00	72. 56	45. 98	44. 06
Listeria	45. 21	44. 83	43. 73	72. 56	100. 00	44. 62	48. 85
M. tuberculosis	69. 14	67. 66	70. 52	45. 98	44. 62	100. 00	49. 25
H. pylori	50. 00	49. 62	48. 51	44. 06	48. 85	49. 25	100. 00

Supplement Figure S1. Sequence comparison of MinD of different bacteria (A) and their similarity percentage analysis (B) using Clustal Omaga software. MinD conserves well and there is about 43% identity between *B. subtilis* and *P. aeruginosa***.**

A.

```
E. coli          -------msntpielkgssftlsvvhlheaepkvihqaledkiaqapaflkhapvvlnvs 53
K. pneumoniae    -------msntpielkgssftlsvvhlhdanpevirqaledkiaqapaflrhapvvvnia 53
P. aeruginosa    msqadlldqdpvfqlkgsmlavtilelahndlarlerqladkvaqapnffrdtplvmald 60
B. subtilis      ----mktkkqqyvtikgtkng---ltlhlddacsfdelldg----lqnml-------sie 42
Listeria         --------mkknvqikgtksg---isiflsdkasiselqqe----lsqll-------adq 38
M. tuberculosis  -------mspspidlkgsnftlsvlhlnnedpqiieqavrekvsqapaffqnapvvlnvs 53
H. pylori        ----mlktnqknvha---------feiekqepeavmefleknhallqyfl-------iif 40
                             .           . :   :   . .           ::

E. coli          aledpv---nwsamhkavsatglrvigvsgckdaqlkaeiekmglpilteg-kek----- 104
K. pneumoniae    sieeev---ewraineaiaatglrimgvsgckiprlkteidragiplleteg-kek----- 104
P. aeruginosa    klpegegrldlpallevyrrhglrtlairagreediaaa-qaldlpvlppsgarerpldi 119
B. subtilis      qytdgkgq-----kisvhvklgnrfl--ykeqeeqlteliaskkdlfvhsi--------- 86
Listeria         kqnpysge-----klevqvqignrlf--seeeereistiihnnsqmkisaf--------- 82
M. tuberculosis  qissga---nlkiicravtdaglrivgisgcqdekmrrvatrsglpllneg-ksa----- 104
H. pylori        ky---------------------------diepevkailrkhqllfletn----rtlng 68
                                                    :           :

E. coli          ---apr--------------papapqa-ptqnttpvtktrlidtpvrsgqriyapqcdli 146
K. pneumoniae    ---asr--------------papsdptppppvasqitktrlidqpvrsgqriyaphcdli 147
P. aeruginosa    kdstprkpaeepspsagearpepak-aeekpaepvsrptkvvktpvrggmqiyaaggdli 178
B. subtilis      -dsevitk-k-----------eaqq-------ireeaeiisvskivrsgqvlqvk-gdll 125
Listeria         -ysnvmsk-d-----------eakk-------wkendqifsmatiirsgqvvqvp-gdfl 121
M. tuberculosis  ---rvaep-ea---------pepetpavssaadsscrktrlinvpvrsgqriyapnsdli 151
H. pylori        rhiktmslkee------tdhakpnh-------sktepkttiyerhirsgeeiysa-nhli 114
                                                              :*.*  :     .::

E. coli          vtshvsagaeliadgnihvygmmrgralagasgdretqifctnlmaelvsiageywlsdq 206
K. pneumoniae    vtnhvsagaeliadgnihvygmmrgralagaggdrdaqifcthlaaelvsiageywlsdn 207
P. aeruginosa    vlaavspgaelladgnihvygpmrgralagvkgdatarifcqqlaaelvsiagnykvaed 238
B. subtilis      ligdvnpggtvraggnifvlgslkgiahagfngnnqaviaasemlptqlrinhvlnrspd 185
Listeria         ligdvnpggqirsngnvfvlgnikgiihagfegdenavvagkflypsqvriagkvygfds 181
M. tuberculosis  vtanvsagaeliadgnihiyglmrgrvlagaagdtesqifcthlhaelisiagqywlsdq 211
H. pylori        flgnihngakiisegcvsvygvcegaivcf----------gerlilkevksaqivfqnki 164
                 .   :  *. : * : * .*   .              :    :

E. coli          ip-aefygkaarlqlvenaltv--qpln---------------- 231
K. pneumoniae    ip-aefygkaarlrlgesalai--qpln---------------- 232
P. aeruginosa    lrrspqwgkavhvslsgdvlni--trl----------------- 263
B. subtilis      ---hiqkgnemecayldtdgnmvierlqhlahlrpdltrleggm 226
Listeria         edykevadtdlfsafvndageivideihkirkirpeisnfqggr 225
M. tuberculosis  ip-gefagkaaklslvnnelti--nnli---------------- 236
H. pylori        ls-----lkeverllvnkniki-itknddildikevl------- 195
                         .            :
```

B.

E. coli	100.00	75.76	38.16	18.69	17.59	58.87	21.69
K. pneumoniae	75.76	100.00	37.99	18.69	18.59	56.90	21.08
P. aeruginosa	38.16	37.99	100.00	23.30	20.98	39.06	21.86
B. subtilis	18.69	18.69	23.30	100.00	36.94	17.91	15.82
Listeria	17.59	18.59	20.98	36.94	100.00	17.82	22.29
M. tuberculosis	58.87	56.90	39.06	17.91	17.82	100.00	17.65
H. pylori	21.69	21.08	21.86	15.82	22.29	17.65	100.00

Supplement Figure S2. Sequence comparison of MinC of different bacteria (A) and their similarity percentage analysis (B) using Clustal Omaga software. MinC shares only about 23% identity between *B. subtilis* and *P. aeruginosa*.

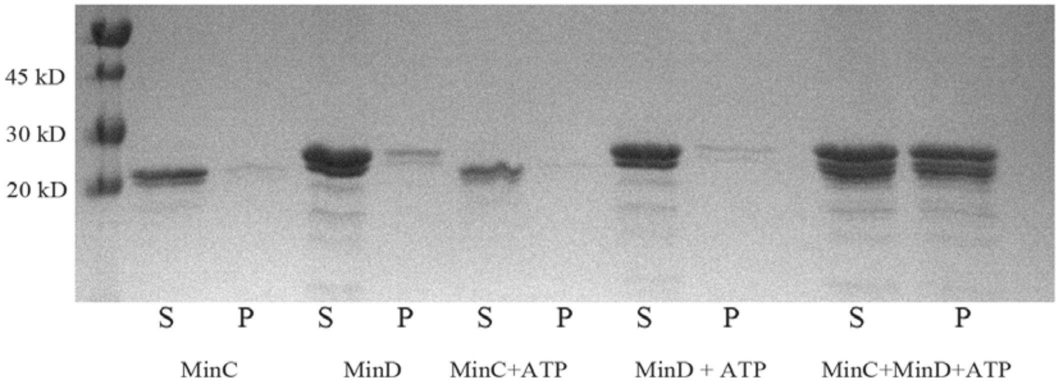

Supplement Figure S3. SDS-PAGE analysis of co-sedimentation of BsMinC and BsMinD proteins. After centrifugation at 50,000 rpm at 20 minutes, no protein were observed in the pellet for BsMinC and BsMinD alone. For the mixture of BsMinC and BsMinD with ATP, the ratio of MinC and MinD of the pellet is 1:1. The concentration of BsMinC and BsMinD is 10 μM and ATP is 1 mM.
